# Supplementary material for: Comparing and modelling land use organization in cities
Source: R Soc Open Sci. 2015 Dec 2;2(12):150449. doi: 10.1098/rsos.150449 (PMC4807451; doi:10.1098/rsos.150449)
Supplement: Supplementary Material for Comparing and modeling land use organization in cities [file rsos150449supp1.pdf]

Supplementary Material for
Comparing and modeling land use organization in cities

Maxime Lenormand[1], Miguel Picornell[2], Oliva G. Cantú-Ros[2], Thomas Louail[3,4], Ricardo Herranz[2], Marc Barthelemy[3,5], Enrique Frías-Martínez[6], Maxi San Miguel[1], and José J. Ramasco[1]

[1]Instituto de Física Interdisciplinar y Sistemas Complejos IFISC (CSIC-UIB), 07122 Palma de Mallorca, Spain
[2]Nommon Solutions and Technologies, Calle de Diego de Leén 47, 28006 Madrid, Spain
[3]Institut de Physique Théorique, CEA-CNRS (URA 2306), F-91191, Gif-sur-Yvette, France
[4]Géographie-Cités, CNRS-Paris 1-Paris 7 (UMR 8504), 13 rue du four, FR-75006 Paris, France
[5]Centre d'Analyse et de Mathématique Sociales, EHESS-CNRS (UMR 8557), 190-198 avenue de France, FR-75013 Paris, France
[6]Telefónica Research, 28050 Madrid, Spain

1 Case studies

In this study, we focused on the five biggest metropolitan areas of Spain, Madrid, Barcelona, Valencia, Seville and Bilbao (Figure S1). These metropolitan areas are very different in terms of sizes and populations (Table S1). For all cities we have selected as urban area the one served by public transportation (bus and metro) instead of the official definition that in the case of Seville includes a much larger extension relatively depopulated.

Table S1: Summary statistics on the metropolitan areas

Metropolitan area	Number of municipalities	Number of inhabitants	Area (km^2)
Madrid	27	5,512,495	1,935.97
Barcelona	36	3,218,071	634
Valencia	43	1,549,855	628.81
Sevilla	8	983,852	352
Bilbao	34	908,916	500.2

2 Data pre-processing

Mobile phone records of anonimyzed users during 55 days (hereafter noted T) within the period of September-November 2009 were aggregated in two different ways. The aggregated data corresponds to the number of users per hour and per base transceiver stations (BTSs) identified with UTM (WSG84) coordinates. A user may appear connected to more than one BTS within a period of one hour. To avoid over counting people the following criteria was used when aggregating the data: each person shall count only once per hour. If a user is detected in k different positions

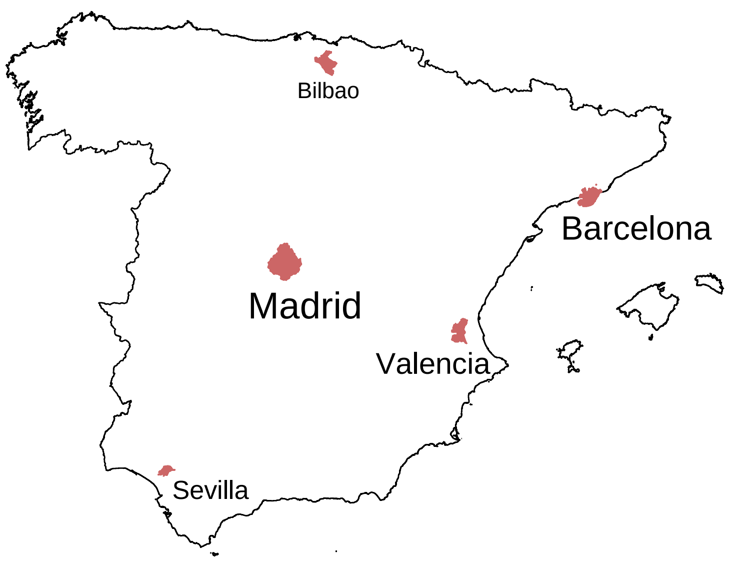

Figure S1: **Map of the metropolitan areas.**

within a certain 1-hour time period, each registered position will count as $(1/k)$ "units of activity". From this aggregated data activity per BTS and per hour is calculated for each day. In order to compute the number of mobile phone users $P_{g,d}(h)$ in a grid cell g (dimension 0.5×0.5 km^2) for a day $d \in T$ between h and $h+1$, where $h \in |[0, 23]|$, we first computed the Voronoi cells associated with each BTS.

2.1 Voronoi cells

First we remove the BTSs with zero mobile phone users and we compute the Voronoi cells associated with each BTSs of the metropolitan area (hereafter called MA). We remark in Figure S2A that there are four types of Voronoi cells:

1. The Voronoi cells contained in MA.
2. The Voronoi cells between MA and the territory outside the metropolitan area.
3. The Voronoi cells between MA and the sea (noted S).
4. The Voronoi cells between MA, the territory outside the metropolitan area and the sea.

To compute the number of users associated with the intersections between the Voronoi cells and MA we have to take into account these different types of Voronoi cells. Let m be the number of Voronoi cells (ie BTSs), $N_{v,d}(h)$ the number of users in a Voronoi cell v (on day d at time h) and A_v the area of v, $v \in |[1, m]|$. The number of users $N_{v \cap MA,d}(h)$ in the intersection between v and MA is given by the following equation:

$$N_{v \cap MA,d}(h) = N_{v,d}(h) \left(\frac{A_{v \cap MA}}{A_v - A_{v \cap S}} \right) \tag{1}$$

We note in Equation 1 that we have removed the intersection of the Voronoi area with the sea, indeed, we assume that the number of users calling from the sea are negligible. Now we consider the number of mobile phone users $N_{v,d}(h)$ and the associated area A_v of the Voronoi cells intersecting MA (Figure S2B).

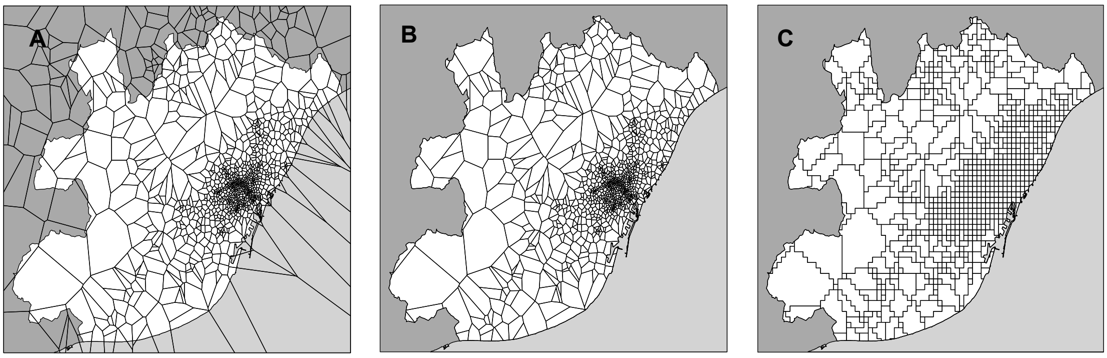

Figure S2: **Map of the metropolitan area of Barcelona.** The white area represents the metropolitan area, the dark gray area represents territory surrounding the metropolitan area and the light grey area represents the sea. (A) Voronoi cells of the mobile phone antennas point pattern. (B) Intersection between the Voronoi cells and the metropolitan area. (C) Recording sites composed of grid cells of dimension 0.5×0.5 km^2.

2.2 Grid cells

Let n be the number of grid cells, the number of mobile phone users $N_{g,d}(h)$ (on day d at time h) is given by the following equation, ∀ g ∈ $|[1, n]|$:

$$N_{g,d}(h) = \sum_{v=1}^{m} N_{v,d}(h) \frac{A_{v \cap g}}{A_v}. \quad (2)$$

Then the set of days T is divided into subsets $T_w \subset T$ and the average number of mobile phone users is computed for each day of the week w (Equation 3).

$$N_{g,w}(h) = \frac{\sum_{d \in T_w} N_{g,d}(h)}{|T_w|} \quad (3)$$

The average number of mobile phone users for the metropolitan areas according to the time and the day of the week are plotted on Figure S3. The profile curve shows two peaks, one peak around 12AM and an other one around 7PM. It also shows that the number of mobile phone users is higher during weekdays than during weekend.

N is normalized such that the total number of users at a given time on a given day is equal to 1, Equation 4,

$$\hat{N}_{g0,w}(h) = \frac{N_{g0,w}(h)}{\sum_{g=1}^{n} N_{g,w}(h)} \quad (4)$$

This normalization allows for a direct comparison between sources with different absolute user's activity. For a given grid cell $g = g_0$ we defined the temporal distribution of users $\hat{N}_{g_0}$ as the concatenation of the temporal distribution of users associated with each day of the week. For each grid cell we obtained a temporal distribution of users (also called signal) represented by a vector of length 24×7. It is possible that some grid cells have exactly the same signal because some Voronoi cells may contain several cells, in this case the grid cells have been aggregated (Figure S2C).

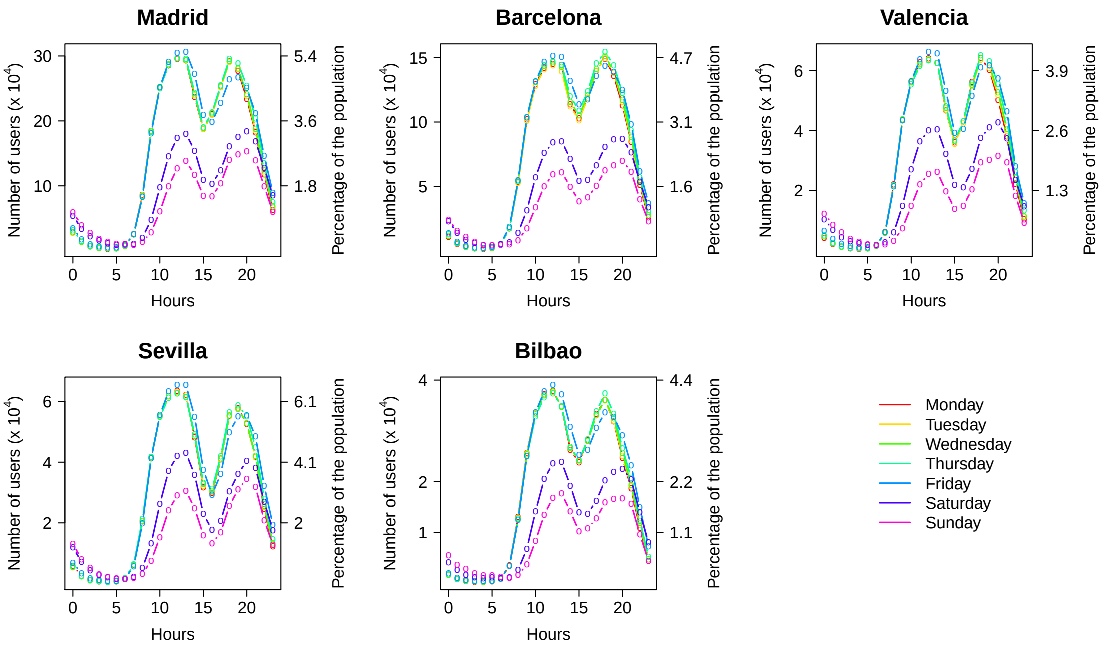

Figure S3: **Average number of mobile phone users per hour according to the day of the week for the five metropolitan areas.**

3 Functional network

3.1 Choice of δ

In the method used to extract the functional network from the mobile phone data presented in the main text we apply a threshold δ to the correlation matrix in order to remove the noise and negative correlations from the correlation matrix. Hence, we have to choose a value of δ high enough to remove the noise but not too high in order to preserve the structure and the properties of the network. Figure S4 displays the distribution of the weights (i.e. correlation coefficient) for the five case studies. One can observe that these distributions can be approximated by a Gaussian distribution. Therefore, we have decided to keep only edges with a weight higher than the weight distribution's standard deviation. In Figure S4 we note that for δ lower than the weight distribution's standard deviation (around 0.4, see details in Table S2) the number of connected components is equal to 1.

Table S2: Statistical properties of the functional networks

City	SD	N	E	$<k>$	$<k>/N$	C	L	C_r	L_r
Madrid	0.42	1,381	222,227	321.8	0.233	0.69	2.04	0.31	1.77
Barcelona	0.38	652	46,573	142.9	0.219	0.62	2.02	0.29	1.79
Valencia	0.35	351	13,847	78.9	0.225	0.66	2.06	0.31	1.84
Sevilla	0.38	188	3,700	39.2	0.209	0.62	2.15	0.26	1.81
Bilbao	0.35	267	8,915	66.8	0.25	0.67	2.03	0.39	1.76

Table S2 summarizes the statistical properties of the functional networks obtained for the five

case studies. In these tables we can observe the threshold (SD), the number of nodes (i.e number of cells) (N), the number of edges (E), the average degree ($< k >$), the average clustering coefficient (C) and the average shortest path length (L). The average clustering coefficient C_r and the average shortest path length L_r have been obtained with a randomly rewired network preserving the degree of the original network by permuting links (4 x (number of edges) times) [1]. We observe that the five networks are very similar, characterized by a high clustering coefficient and low average shortest path.

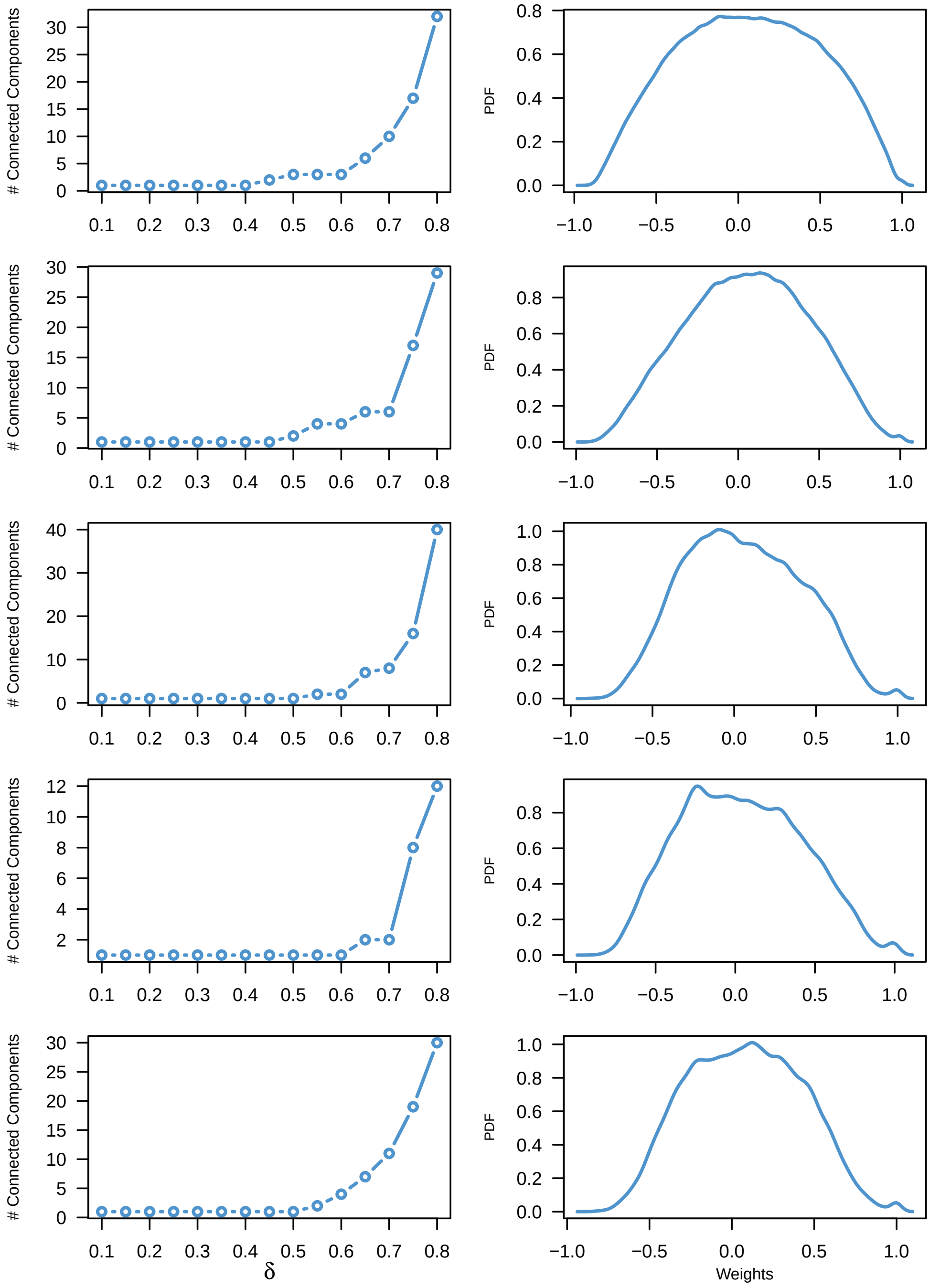

Figure S4: **Number of connected components as a function of δ (Left) and weight distribution (Right) for the five case studies.** From top to bottom, Madrid, Barcelona, Valencia, Sevilla and Bilbao.

3.2 Community detection

Community detection in complex networks has recently been the subject of an abundant literature and a large number of algorithms has been proposed the last few years. The purpose of these algorithms is to identify closely connected groups of nodes within a network. To do so, several techniques are used such as maximizing the modularity, measuring probability flows of random walks or optimizing the local statistical significance of communities.

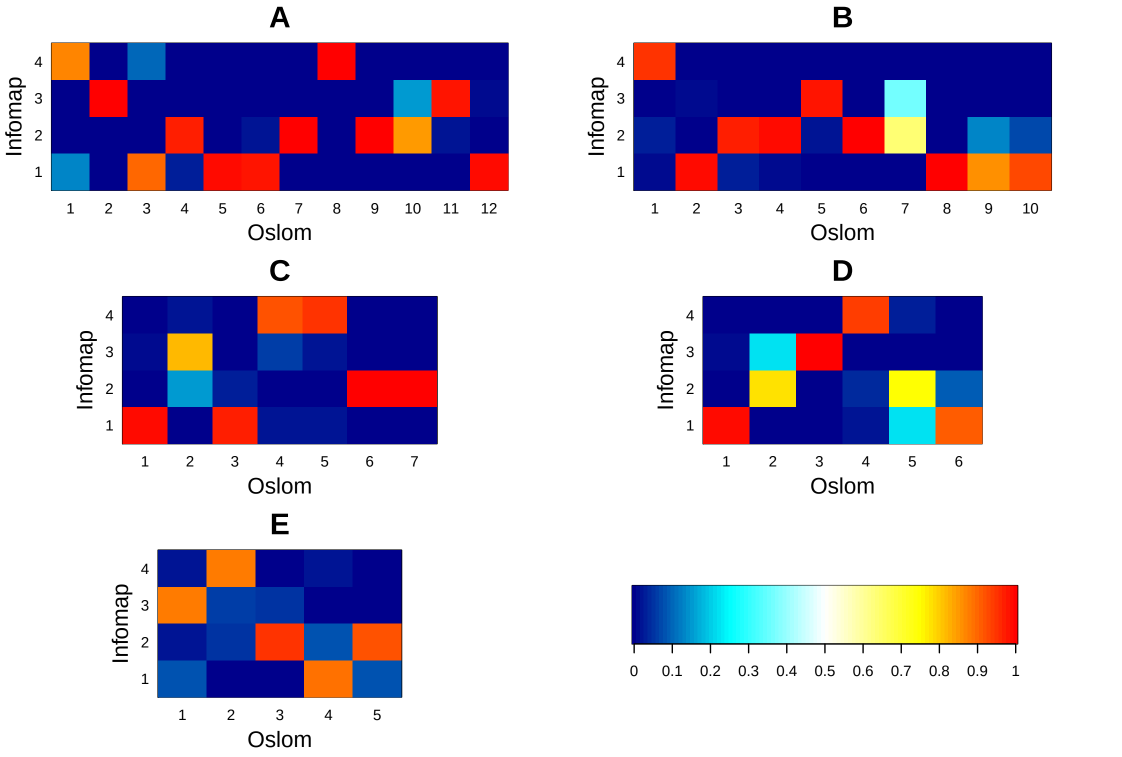

Figure S5: **Contingency tables between the partitions obtained with Infomap and OSLOM for each case study.** (A) Madrid. (B) Barcelona. (C) Valencia. (D) Sevilla. (E) Bilbao. Each row represents a cluster obtained with Infomap and each column represents a cluster obtained with OSLOM. The matrices have been normalized so that the sum of each column is equal to one.

In this paper, we have decided to use the Infomap method proposed in [2]. Infomap finds communities by using the probability of flow of random walks on the network as a proxy for information flow in the real system and then decompose the graph into groups of nodes among which information flows easily. As shown in [3], this method gives good results, however, to evaluate the robustness of the results, the analysis has also been performed with two other clustering methods, Oslom [4, 5] and Louvain [6]. Oslom is a method based on a topological approach to detect statistically significant cluster whereas Louvain is based on modularity optimization which means finding the optimal partition maximizing the density of links within clusters and minimizing the density of links between clusters.

In order to compare the partition obtained with the different method we have plotted in Figure S5 and S6, respectively, the contingency tables between the partitions obtained with Infomap and Oslom and Infomap and Louvain for each case study. In these figures, each plot represents a contingency table C in which each element C_{ij} is the number of nodes which belong to the cluster i detected with Infomap and to the cluster j detected with Oslom or Louvain. The matrices have been normalized so that the sum of each column is equal to one. This normalization allows us

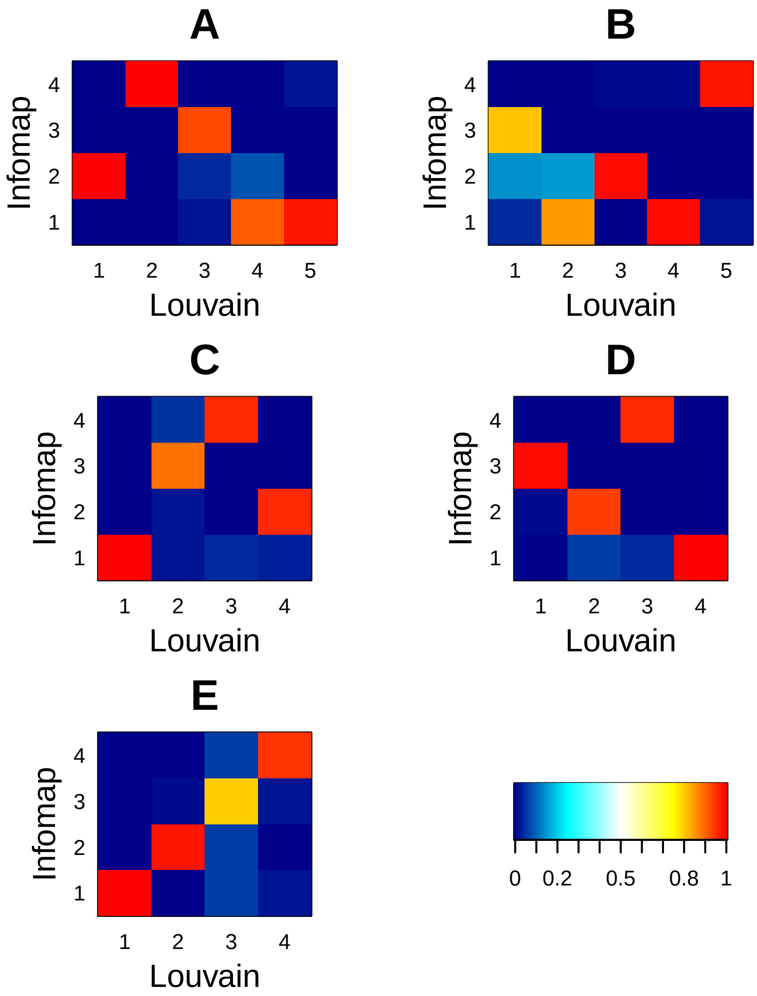

Figure S6: **Contingency tables between the partitions obtained with Infomap and Louvain for each case study.** (A) Madrid. (B) Barcelona. (C) Valencia. (D) Sevilla. (E) Bilbao. Each row represents a cluster obtained with Infomap and each column represents a cluster obtained with Louvain. The matrices have been normalized so that the sum of each column is equal to one.

to study how the nodes belonging to the groups obtained with Oslom or Louvain are distributed among the clusters found with Infomap. First, we can observe that the number of communities detected with Louvain or Oslom is always greater or equal to the ones obtained with Infomap. Indeed, Louvain has detected a similar number of clusters whereas the number of communities detected with Oslom increases with the size of the metropolitan area, from 5 clusters for Bilbao to 12 for Madrid. However, it is worth noting that in most of the cases, more than 80% of the nodes belonging to the Oslom and Louvain's clusters are gathered in one Infomap cluster. This means that even if the size of the partitions are different, we observe that clusters obtained with Louvain and Oslom are sub-clusters of clusters identified with Infomap.

4 Comparison with cadastral data

In order to validate the results we compared the land use patterns obtained with our algorithm with cadastral data available on the Spanish Cadastral Electronic Site[1]. The dataset contains information about land use for each cadastral parcel of the metropolitan area of Madrid and Barcelona (about $650,000$ parcels). In particular, we have for each cadastral parcel the net internal area devoted to Residential, Business and Industrial uses. We can use these data to identify the dominant cadastral land use in each grid cell classified as Residential, Business and Industrial uses by the community detection algorithm. To do so we need to define a rule to determine what is the dominant land use in a cell. Intuitively, one would tend to identify the dominant land use in a cell as the land use class with the largest area. However, Residential use is the land use class with the largest area in most of the cell leading to an over-representation of Residential cells in the metropolitan area. To circumvent this limitation we introduce two thresholds δ_{Bus} and δ_{Log} to identify Business and Logistics cells with cadastral data. If the fraction of area devoted to Business in a grid cell is higher than δ_{Bus} then the grid cell is classified as Business. Otherwise, if the fraction of area devoted to Logistics is higher than δ_{Log} then the grid cell is classified as Industry. Finally, if the fraction of area devoted to Business and Logistics is, respectively, lower than δ_{Bus} and δ_{Log} then the grid cell is classified as Residential.

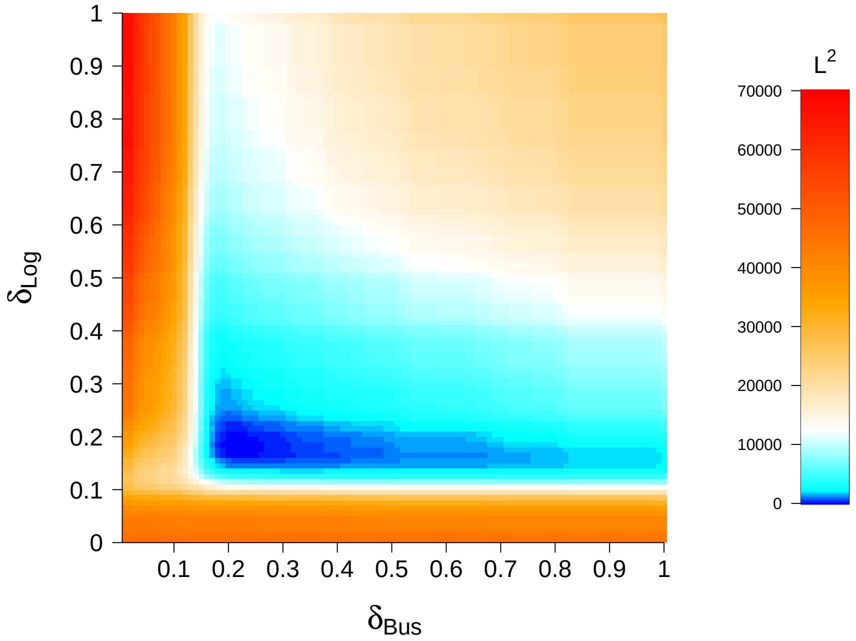

Figure S7: **Comparison with cadastral data**. L^2 distance between the distribution of the fraction of cells according to the land use type (Residential, Business and Logistics) obtained with our algorithm and the cadastral data as a function of δ_{Bus} and δ_{Log} for the municipality of Barcelona.

Hence, we can adjust the values of these two thresholds in order to obtain a distribution of the fraction of cells according to the land use type similar to the one obtained with our algorithm. To this end we have calibrated these parameters by minimizing the L^2 distance between the distribution of the fraction of cells according to the land use type obtained with the cadastral data and the one obtained with our algorithm for the municipality of Barcelona which represents 20% of the

[1] http://www.sedecatastro.gob.es/OVCInicio.aspx

metropolitan area of Barcelona. In Figure S7, we can observe that the minimum is reached for $\delta_{Bus} = 0.2$ and $\delta_{Log} = 0.2$. Now we can use these values to identify the dominant cadastral land use in each grid cell of the metropolitan area of Barcelona and Madrid.

Table S3: Confusion matrix of the classification for Madrid and Barcelona. For the Residential, Business and Logistics rows and columns, the value in the ith row and the jth column gives the percentage of grid cells classified as use i by the cadastral classification which are classified as belonging to the class j by the algorithm. The Total is the distribution of the percentage of cells according to the land use type obtained with our algorithm (row) and the cadastral data (column) with the threshold values $\delta_{Bus} = 0.2$ and $\delta_{Log} = 0.2$.

Madrid

Cadastral \ Algorithm	Residential	Business	Logistics	Total
**Residential**	**71.23**	21.67	7.1	49.04
**Business**	30.84	**62.33**	6.83	39.55
**Logistics**	22.9	32.06	**45.04**	11.41
**Total**	49.74	40.42	9.84	100

Barcelona

Cadastral \ Algorithm	Residential	Business	Logistics	Total
**Residential**	**68**	26.55	5.45	47.5
**Business**	28.99	**52.17**	18.84	35.75
**Logistics**	13.4	32.99	**53.61**	16.75
**Total**	45.77	37.65	16.58	100

We find a percentage of correct predictions equal to 65% for Madrid and 60% for Barcelona which is consistent with values obtained in other studies, 54% in [7] and 58% in [8]. Furthermore, for both case studies, almost all land use types have a percentage of correct predictions higher than 50% (Table S3).

5 Calibration of γ

The value of γ was calibrated in order to reproduce the evolution of the entropy index as a function of the number of divisions by side obtained with the data. We chose the value of γ minimizing the Euclidean distance between the observed values and the average values obtained with the model with 100 replications. The best results have been obtained with the value $\gamma = 0.8$ (Figure S8).

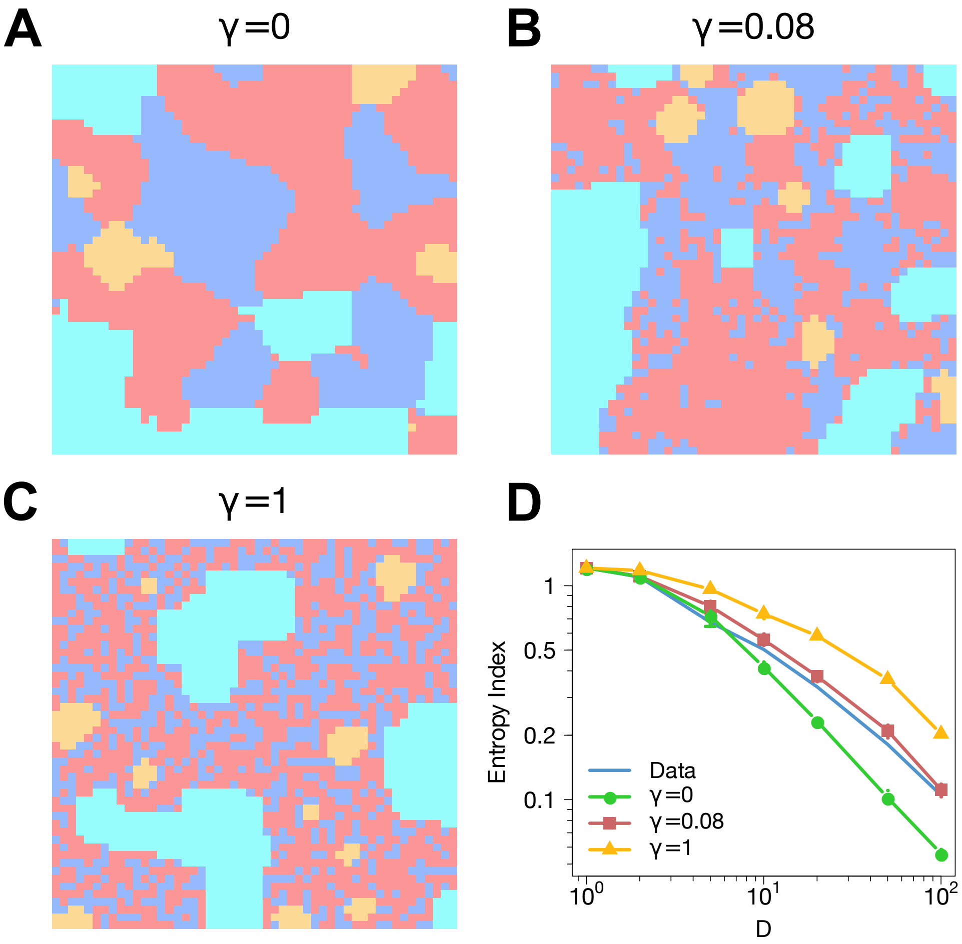

Figure S8: **Calibration of γ**. Results obtained with different values of γ. (A) $\gamma = 0$; (B) $\gamma = 0.8$; (C) $\gamma = 1$. The 2D lattice used to represent the urban space is composed of $50 \times 50 = 2,500$ cells. The model seems to converge after $300,000$ iterations but to ensure the convergence all the results shown in the paper were obtained with $500,000$ iterations. (D) Average entropy index as function of the lateral number of divisions (inverse scale) D according to γ.

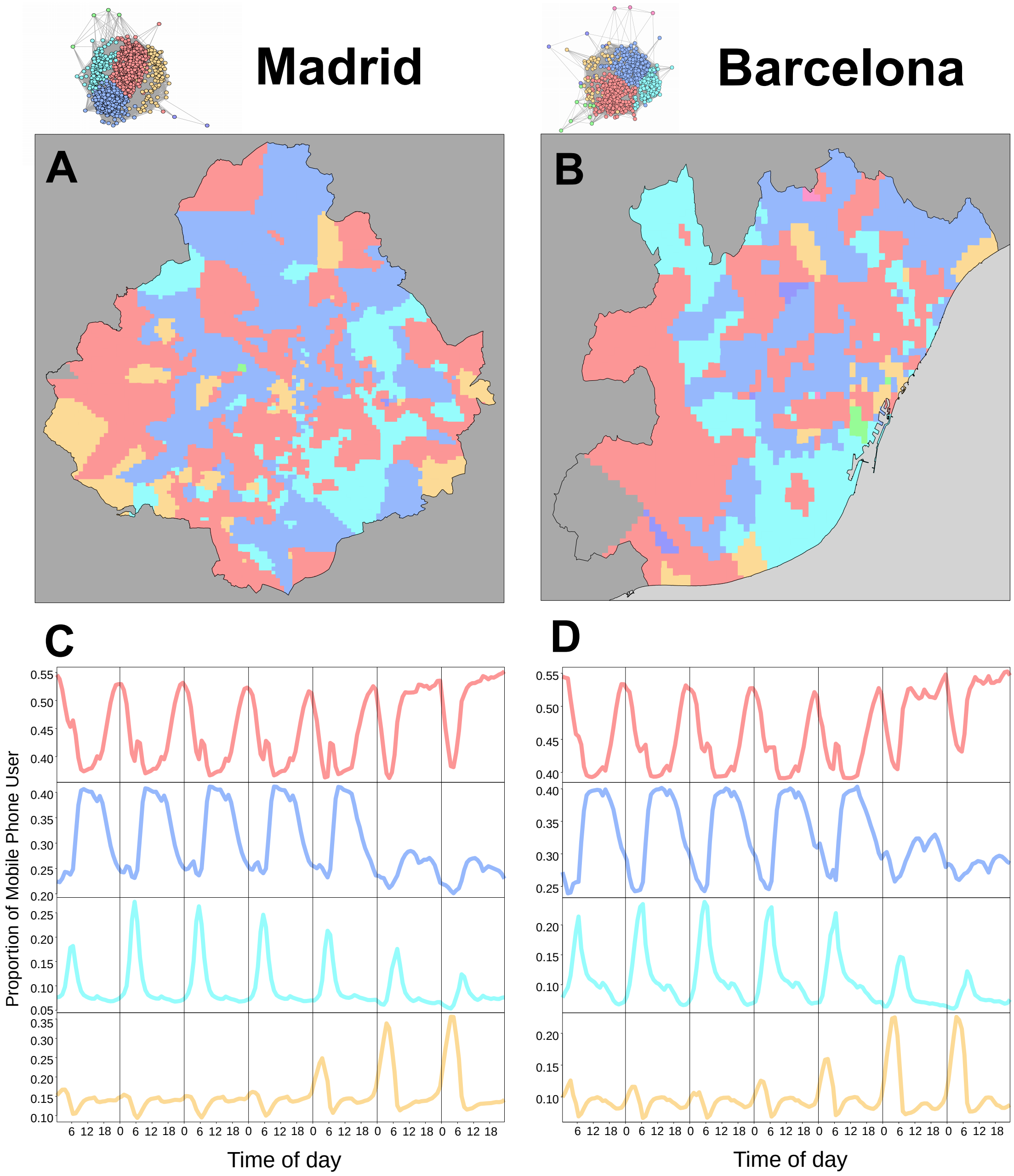

Figure S9: (A-B) Geographical location of the clusters for Madrid and Barcelona. (C-D) Temporal patterns associated with the four clusters for both metropolitan areas. In red, Residential cluster; In blue, Business; In cyan, Logistics; And in orange, Nightlife.

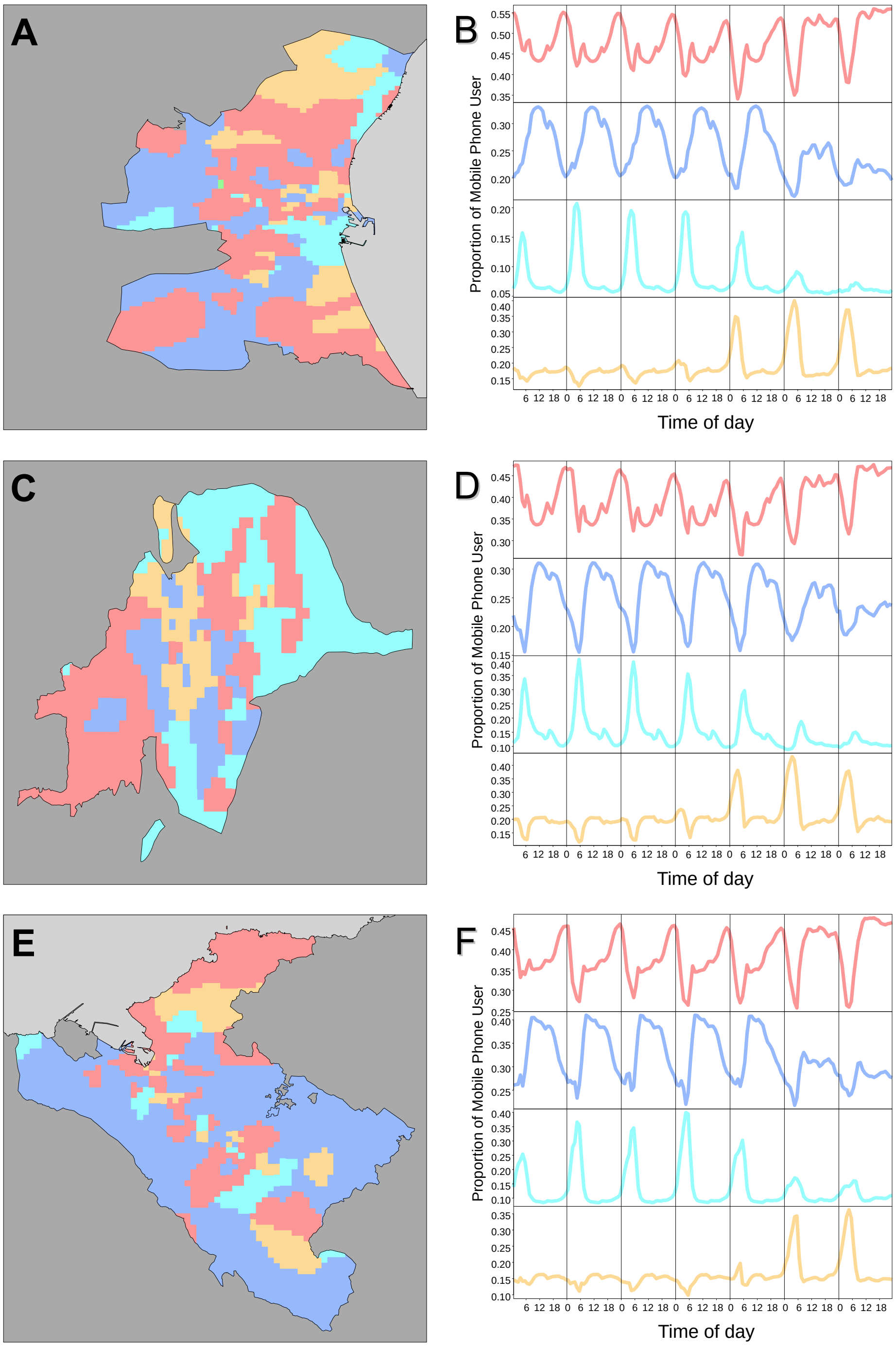

Figure S10: (A), (C) and (E) Geographical representation of the communities for Valencia (A), Sevilla (C) and Bilbao (E). (B), (D) and (F) Temporal patterns associated with the communities for the metropolitan area of Valencia (B), Sevilla (D) and Bilbao (F). In red, the Residential community; In blue, the Business community; In cyan, the Logistics/Industry community; In orange, the Nightlife community.

References

[1] Maslov S, Sneppen K. 2002 Specificity and stability in topology of protein networks. *Science* **296**, 910–913. (doi:10.1126/science.1065103)

[2] Rosvall M, Bergstrom CT. 2008 Maps of random walks on complex networks reveal community structure. *Proceedings of the National Academy of Sciences* **105**, 1118–1123. (doi:10.1073/pnas.0706851105)

[3] Lancichinetti A, Radicchi F, Fortunato S. 2009 Community detection algorithms: a comparative analysis. *Physical Review E* **80**, 056117. (doi:http://dx.doi.org/10.1103/PhysRevE.80.056117)

[4] Lancichinetti A, Radicchi F, Ramasco JJ. 2010 Statistical significance of communities in networks. *Physical Review E* **81**, 046110. (doi:10.1371/journal.pone.0018961)

[5] Lancichinetti A, Radicchi F, Ramasco JJ, Fortunato S. 2011 Finding Statistically Significant Communities in Networks. *PLoS ONE* **6**, e18961+. (doi:10.1371/journal.pone.0018961)

[6] Blondel V, Guillaume J, Lambiotte R, Mech E. 2008 Fast unfolding of communities in large networks. *J. Stat. Mech* P10008. (doi:10.1088/1742-5468/2008/10/P10008)

[7] Toole JL, Ulm M, González MC, Bauer D. 2012 Inferring Land Use from Mobile Phone Activity. In *Procs. of the ACM SIGKDD International Workshop on Urban Computing*, pp. 1–8. (doi:10.1145/2346496.2346498)

[8] Pei T, Sobolevsky S, Ratti C, Shaw SL, Zhou C. 2014 A new insight into land use classification based on aggregated mobile phone data. *International Journal of Geographical Information Science* **28**, 1988–2007. (doi:10.1080/13658816.2014.913794)
